# Weather-Domain Transfer-Based Attention YOLO for Multi-Domain Insulator Defect Detection and Classification in UAV Images

**DOI:** 10.3390/e26020136

**Published:** 2024-02-01

**Authors:** Yue Liu, Xinbo Huang, Decheng Liu

**Affiliations:** 1 School of Electrical Engineering, Xi’an University of Technology, Xi’an 710054, China; yliu@stu.xaut.edu.cn; 2 School of Electronics and Information, Xi’an Polytechnic University, Xi’an 710048, China; 3 School of Cyber Engineering, Xidian University, Xi’an 710126, China; dchliu@xidian.edu.cn

**Keywords:** cross-modality information, insulator defect detection, classification, image processing

## Abstract

Insulator defect detection of transmission line insulators is an important task for unmanned aerial vehicle (UAV) inspection, which is of immense importance in ensuring the stable operation of transmission lines. Transmission line insulators exist in complex weather scenarios, with small and inconsistent shapes. These insulators under various weather conditions could result in low-quality images captured, limited data numbers, and imbalanced sample problems. Traditional detection methods often struggle to accurately identify defect information, resulting in missed or false detections in real-world scenarios. In this paper, we propose a weather domain synthesis network for extracting cross-modality discriminative information on multi-domain insulator defect detection and classification tasks. Firstly, we design a novel weather domain synthesis (WDSt) module to convert various weather-conditioned insulator images to the uniform weather domain to decrease the existing domain gap. To further improve the detection performance, we leverage the attention mechanism to construct the Cross-modality Information Attention YOLO (CIA-YOLO) model to improve the detection capability for insulator defects. Here, we fuse both shallow and deep feature maps by adding the extra object detection layer, increasing the accuracy for detecting small targets. The experimental results prove the proposed Cross-modality Information Attention YOLO with the weather domain synthesis algorithm can achieve superior performance in multi-domain insulator datasets (MD-Insulator). Moreover, the proposed algorithm also gives a new perspective for decreasing the multi-domain insulator modality gap with weather-domain transfer, which can inspire more researchers to focus on the field.

## 1. Introduction

**Figure 1:**
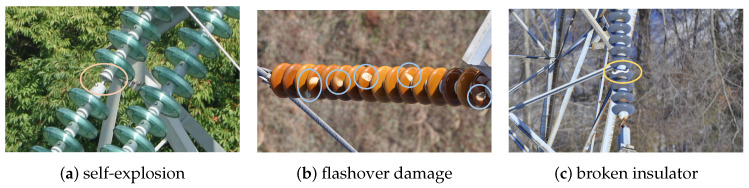
Appearance overview of insulator defects. (**a**) Self-explosion. (**b**) Flashover damage. (**c**) Broken insulator.

The high-voltage transmission lines are mainly distributed in geographical environments such as mountains, forests, grasslands, farmlands, and fields. It is easy for insulators to suffer various levels of damage under adverse weather conditions. The three insulator defects of transmission lines are th self-explosion insulator defect, the flashover insulator defect, and the broken insulator defect as shown in Figure 1. Insulators are an important component of transmission lines, playing an important role in mechanical support and electrical insulation. During operation, they withstand vertical loads on conductors, horizontal tension, as well as the impact of weather and chemicals, resulting in varying degrees of damage and posing potential safety hazards to the stability of transmission line operation [1]. In severe cases, these defects can cause power grid failures in various regions, leading to significant economic losses. Liu et al. [2] pointed out that the detection of power line components and their common faults is a fundamental task in the field of power line detection, and it is also one of the most popular research topics. Therefore, discovering and replacing damaged insulators on time can ensure the effective and normal operation of transmission lines. With the development of smart grids and image detection technologies [3,4], traditional manual inspection methods for insulator patrol have gradually been replaced by low-cost, efficient, and flexible UAV inspection [5,6].

The inspection method based on drones utilizes drones to capture aerial images of insulators and then analyze and process the collected image information. However, many existing methods only focus on insulator defect detection in a single weather condition, ignoring the impact of weather changes on the information of insulator images collected by unmanned aerial vehicles (UAVs). This leads to poor detection results in real-world scenarios. In this case, it is of great significance for the safe operation of the power system to study the detection algorithm for multi-domain insulator defects based on UAV for transmission line insulation defects. Therefore, this paper proposes a weather domain synthesis network for extracting cross-modality discriminative information on multi-domain insulator defect detection and classification tasks to mimic real-world scenarios.

The main contributions of our paper can be summarized as follows:

1. We explore a novel weather domain transfer-based framework for multi-domain insulator defect detection and classification tasks, which gives a new perspective to decrease the multi-domain insulator modality gap in diverse weather conditions.
2. The proposed Cross-modality Information Attention YOLO module is designed to leverage attention mechanisms and add detection layers in the network head for small targets, which can improve the model’s detection performance on multi-domain insulator defects.
3. We constructed a new multi-domain insulator dataset (MD-Insulator) for defect detection and classification. The self-built dataset contains 16,430 insulator images and three different defect detection categories, namely self-explosion defects, flashover damages, and insulator broken defects. The MD-Insulator dataset also includes insulator images under complex weather conditions, such as rainy, foggy, and snowy, to simulate multi-domain insulators, which can enhance the model’s detection performance for insulators under multi-domain weather conditions.
4. The experimental results of what we proposed, the multi-domain insulator dataset (MD-Insulator), illustrate the superior performance of the proposed method compared with the comparison methods.

The remainder of this article is organized as follows. Section 2 provides a brief overview of the current state of insulator defect detection and the system frameworks. In Section 3, we describe in detail the weather domain synthesis (WDSt) module and the Cross-modality Information Attention YOLO (CIA-YOLO) module. Section 4 presents the experimental results and analysis. Conclusions are drawn in Section 5.

## 2. Related Work

Liu et al. [1] innovatively classify insulation defect detection methods into two categories: multi-task and sequential task strategies based on the steps of the model task. Multi-task strategies’ detection network is directly on the input insulation image using target detection algorithms, and the advantages are fast detection speed and relatively simple network models. However, they are not good for detecting small targets. Sequential task strategies first locate the insulation from the input image and then locate the defects on the located insulation image. Although it can accurately detect small targets and improve the defect detection results, its detection method is more complex and requires a large amount of computational resources for training. The most important thing is that the results of image segmentation will directly affect the accuracy of defect detection. To solve these problems, researchers have conducted extensive research on defect detection. In recent years, the most common insulation detection method based on UAVs inspection is the target detection method improved based on Faster Region-CNN (Faster RCNN) [7,8,9,10,11] and the You Only Look Once (YOLO) [12,13,14,15] network. Gao et al. [10] combined a batch normalization convolutional fast attention model (BN-CBAM) with a feature fusion module to improve the detection performance of small insulation targets and insulation defects. Guo et al.’s [9] Faster R-CNN comprises the depth residual network that is combined with soft non-maximum suppression to simultaneously detect insulation and defects in the original image. Wang et al. [11] added an improved region proposal network (RPN) combined with ResNet for feature extraction to better detect small defects on insulation. Ma et al. [12] proposed a YOLOv4 insulation detection model based on the joint Gaussian distance intersection loss function, which improves the problem of low detection accuracy and slow positioning speed of insulation. In addition, rectifying and repositioning insulation on inclined insulation also significantly improves the accuracy of insulation detection. Liu et al. [13] designed an insulation detection network MTI-YOLO (YOLOtiny for insulation) pyramid pool (SPP) detector for complex aerial images to improve the accuracy and feature expression of specific size insulation. Since the defect area of the insulation occupies a small proportion of the insulation image and is difficult to detect, Zhang et al. [15] proposed to introduce a densely connected feature pyramid network into YOLOv3. Bao et al. [16] added coordinate attention (CA) modules to YOLOv5 to improve the insulation detection results by making the network pay more attention to insulation features and reducing the impact of complex backgrounds on the model.

The YOLO series target detection network is popular because of its fast detection speed. It uses a separate CNN network to directly predict the classification and location of various targets and has made great progress in insulator detection. However, people have only focused on improving the detection accuracy of small targets while ignoring the impact of natural factors on the insulation defect detection model. The various weather conditions and complex backgrounds can result in low-quality images captured of insulators inspected by UAVs, making it more suitable to study multi-domain insulator defect detection and classification in real life. The existing public dataset that can be used for multi-domain insulators was released by Zhang et al. [17] in 2010 with the synthetic fog insulation data (SFID) dataset. This dataset includes 13,718 images of insulators and self-explosion defects under foggy and sunny weather conditions. To further study multi-domain insulator defect detection methods, we collect and construct a multi-domain insulator dataset (MD-insulator), which contains three different insulation defects. Furthermore, we propose a Cross-modality Information Attention YOLO with weather domain synthesis for the multi-domain insulator defect detection and classification model. Firstly, we designed a novel weather domain synthesis (WDSt) module for various weather-conditioned insulator images to the uniform weather domain to decrease the existing domain gap. To further improve the detection performance, we leverage a Cross-modality Information Attention YOLO (CIA-YOLO) model using an attention mechanism and add the extra object detection layer, increasing the accuracy for detecting multi-domain insulator defects. Here, we provide a new perspective for decreasing the multi-domain insulation modality gap of the weather domain transfer, with a detailed algorithm description in the following sections.

## 3. The Proposed Method

With the continuous development of UAV technology [18], UAV inspection has been widely used in various industries. Various weather conditions can cause damage to the insulators of power transmission lines, even leading to failures [19]. Therefore, regular use of UAV inspections to detect insulation defects can effectively and accurately assess the insulation condition.

**Figure 2:**
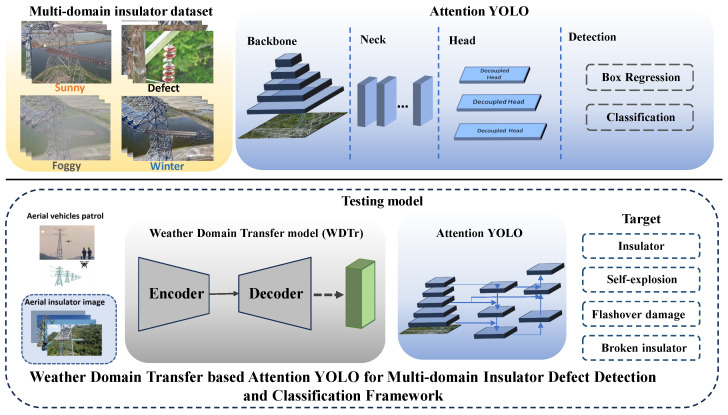
The framework of multi-domain insulator defect detection and classification. In the training model, a Cross-modality Information Attention YOLO (CIA-YOLO) model for multi-domain insulator detection and classification is trained by the Multi-domain insulator dataset (MD-insulator), and we leverage an attention mechanism to the model to improve the detection capability for multi-domain insulator defects; in the testing model, we design a novel weather domain synthesis module (WDSt) to convert various weather-conditioned insulator images to the uniform weather domain to decrease the existing domain gap.

Currently, deep learning-based insulation defect detection models are trained by sunny insulator images from the China Power Line Insulator Subset Dataset (CPLID), which X. Tao et al. [20] proposed in 2018. However, these models are unsuitable for multi-domain weather insulation defect detection tasks. Compared to sunny days, foggy days can result in low-quality insulator images of UAV inspections and often struggle to accurately identify defect targets; snowy days may cover up insulators, and the model fails to detect targets. Additionally, frost weather conditions may condense on the surface of insulators and change their shapes, thus affecting the recognition capabilities of the detection model. Various weather conditions significantly impact the safe operation of power transmission line insulators, so studying multi-domain insulation defect detection for power transmission lines based on UAV inspection has important practical significance for ensuring the safety and stability of power grids. In this paper, we propose a weather domain synthesis network for extracting cross-modality discriminative information on multi-domain insulator defect detection and classification tasks to mimic real-world scenarios. The detection and classification framework is shown in Figure 2.

The model proposed in this paper is divided into two strategies: the training model and the testing model. In the training model, we trained a model for multi-domain insulator defect detection and classification using a multi-domain insulator dataset and further improve the detection performance, where we leveraged attention mechanisms and added detection layers in the network head for small targets. In the testing model, we design a novel weather domain synthesis module (WDSt) to convert various weather-conditioned insulator images to the uniform weather domain to decrease the existing domain gap. The detailed model structure will be illuminated in the following chapters. The proposed Cross-modality Information Attention YOLO with weather domain synthesis for the multi-domain insulator defect detection and classification model can distinguish between three common types of insulator defects: self-explosion, flashover damage, and broken insulator. Moreover, we provide a new perspective for reducing the multi-domain insulator modality gap with weather-domain transfer, which can inspire more researchers to focus on the field.

### 3.1. Motivation

As mentioned above, the challenges of multi-domain insulator defect detection and classification tasks lie in (1) the domain discrepancy, and (2) multi-scale and low-quality insulator images. The former is due to the diverse image capture conditions, and the latter is due to the different properties of cameras in real-world applications. Both of them will bring adverse effects on the insulator defect detection task.

For the existing domain discrepancy, we aim to construct a two-stage evaluation framework to eliminate the large domain gap in diverse weather conditions. Here, we utilize the novel weather domain synthesis module to translate different domain images into the same domain, which will help improve the insulator detection performance.

**Figure 3:**
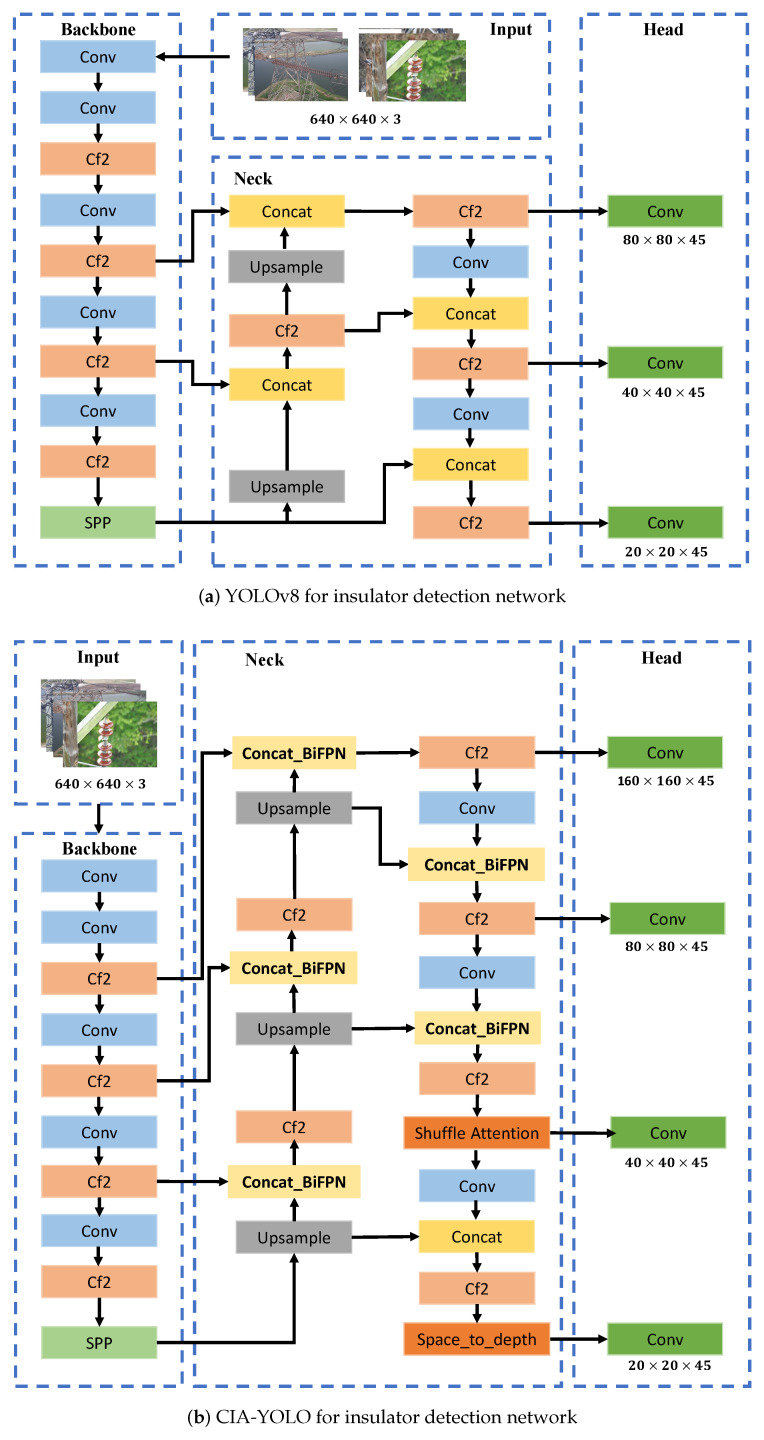
Insulator detection network. (**a**) YOLOv8 for insulator detection network. (**b**) Cross-modality Information Attention YOLO for insulator detection network. We added a new small target detection layer, used a repeated weighted bidirectional feature pyramid network for feature fusion, and introduced an effective attention mechanism to enable the network to accurately focus on insulator defect information with the input, improving the detection accuracy of multi-domain insulators. At the same time, it reduces the model complexity and computational overhead.

Considering the properties of the multi-scale and low-quality images, we aim to design the cross-modality information attention YOLO architecture with better generalization and robustness compared with the original YOLO, as shown in Figure 3. (1) The deeper feature maps make it difficult to learn the feature information of small targets. The proposed method is designed to enhance the detection model’s adaptability to insulator and insulator defect detection in adverse weather conditions. (2) To improve the model’s receptive field and feature extraction capabilities for small targets, we construct the Cross-modality Information Attention YOLO insulator defect detection and classification network and add a small target detection layer in the neck part to splice the shallow feature maps with the deep feature maps, aiming to make the network more focused on the detection of small targets and improve the detection performance of small targets. (3) We improved the feature extraction network by introducing repeated effectively bidirectional cross-scale connections and weighted feature fusion for multi-scale feature fusion, which resulted in a higher detection performance for the model under multi-domain insulators.

### 3.2. Cross-Modality Information Attention YOLO Model for Multi-Domain Insulator Defect Detection and Classification

The overall structure of the trained CA-YOLO algorithm model is shown in Figure 3b. In insulator defect detection tasks, the dataset contains small targets such as self-explosion defects, insulator damage, and flashover defects. In standard object detection, small targets like defects often suffer from missed detections or poor detection performance. The YOLOv8 model has three detection heads that can detect targets with feature map sizes of 80×80, 40×40, and 20×20, covering detection scales of 8×8 and above. However, in insulator defect detection, insulator damage and flashover defects often exist at even smaller scales. Therefore, we have added a 160×160 detection feature map specifically for detecting tiny objects of 4×4 and above. By expanding the modeling framework of the receptive field and further optimizing the backbone feature extraction, we have improved the model’s ability to recognize tiny defects. Multi-domain insulator detection tasks are susceptible to complex scenes and multi-scale information. Currently, the main approach to addressing this issue is through the attention mechanism [21,22,23,24,25,26,27] and weighting optimization. Currently, widely used attention mechanisms include channel attention and spatial attention to enhance the original features.

Some studies, including CBAM [26] and SA [23], integrated spatial attention and channel attention into one module, which has achieved improvements but suffered from either convergence difficulty or heavy computational burden. Other studies have tried to simplify the structure of channel or spatial attention such as ECA-Net [27], which simplifies the process of computing channel weights in SE [24] blocks by using one-dimensional convolutions. Hou et al. [25] proposed embedding location information into channel attention to compensate for the crucial location information that is often overlooked in visual tasks.

The feature pyramid structure has been effectively used for multi-scale feature fusion in object detection. In the YOLOv8 model, PANet is used for multi-scale feature fusion in object detection. However, as image resolution increases and target scenes become more complex, the general FPN and PANet structures may not fully unleash their potential. This is especially true when extracting deeper features, as there is a risk of losing target information and failing to detect objects. We have chosen a novel feature fusion network structure called BiFPN (weighted bidirectional feature pyramid network). BiFPN’s innovative bidirectional cross-scale connections and weighted feature map fusion allow the model to fuse more features without increasing costs, further optimizing the model’s feature extraction. The Bidirectional Feature Pyramid Network (BiFPN) of multi-scale feature fusion is calculated as follows:

(1) $$\begin{array}{cc} P_{i}^{td}= & Conv\left(\frac{\omega_{1}\cdot P_{i}^{in}+\omega_{2}\cdot Resize\left(P_{i+1}^{in}\right)}{\omega_{1}+\omega_{2}+\epsilon }\right),\\ P_{i}^{out}= & Conv\left(\frac{\omega_{1}^{\prime }\cdot P_{i}^{in}+\omega_{2}^{\prime }\cdot P_{i}^{td}+\omega_{3}^{\prime }\cdot Resize\left(P_{i-1}^{out}\right)}{\omega_{1}^{\prime }+\omega_{2}^{\prime }+\omega_{3}^{\prime }+\epsilon }\right). \end{array}$$

Here, $P_{i}^{td}$ is the intermediate feature at level *i* on the top–down pathway, and $P_{i}^{out}$ is the output feature at level *i* on the bottom-up pathway. Resize is usually an upsampling or downsampling op for resolution matching, and Conv is usually a convolutional op for feature processing, $P_{i}^{in}$ represents a feature level with a resolution of 1/2i of the input images, $\omega_{i}\geq 0$, and ϵ=0.0001 is a small value to avoid numerical instability. Finally, we add a lightweight and efficient attention mechanism to enable the network to accurately focus on information related to the detection target. These network improvements achieve higher accuracy while reducing the model complexity and computational overhead.

The shuffle attention (SA) is a lightweight and efficient attention mechanism, which constructs channel attention and spatial attention simultaneously. For a given feature map $X\in R^{C/G\times H\times W}$, where *C*, *H*, *W* indicate the channel number, spatial height, and width, respectively, SA first divides *X* into *G* groups along the channel dimension. The group randomly permutes the input features to perform cross-attention calculations at different computational scales. In multi-domain insulator defect detection tasks, this helps the model better understand and utilize image spatial information and context, improving the model’s expressive ability and enhancing its performance in handling complex scenes and multi-scale information.

The final output of channel attention can be obtained by

(2) $$\begin{array}{c} X_{k1}^{\prime }=\sigma \left(F_{c}\left(s\right)\cdot X_{k1}\right)=\sigma \left(W_{1s}+b_{1}\right)\cdot X_{k1}, \end{array}$$

where $\sigma \left(\cdot \right)=sigmuid\left(\cdot \right)$, $W_{1}\in R^{C/2G\times 1\times 1}$ and $b_{1}\in R^{C/2G\times 1\times 1}$ are parameters used to scale and shift *s*, as well as generate channel-wise statistics as $s\in R^{C/2G\times 1\times 1}$.

The final output of spatial attention is obtained by

(3) $$\begin{array}{c} X_{k2}^{\prime }=\sigma \left(W_{2}\cdot GN\left(X_{k2}\right)+b_{2}\right)\cdot X_{k2}, \end{array}$$

where $W_{2}$ and $b_{2}$ are parameters with shape $R^{C/2G\times 1\times 1}$. The two branches are concatenated to make the number of channels the same as the number of inputs, $X_{k}^{\prime }=\left[X_{k1}^{\prime }\cdot X_{k2}^{\prime }\right]\in R^{C/G\times H\times W}$.

To improve the detection performance of insulator detection models in low-resolution images and small object tasks, we apply the Space-to-depth layer (SPD-Conv) [28] to YOLOv8 to create a new Convolutional Neural Networks (CNN) architecture that reduces the spatial dimension size without losing information while preserving information within the channel, which helps improve the model’s ability to handle more difficult tasks. This approach reduces information loss, improves the accuracy of feature extraction, optimizes the model’s ability to process small objects and low-resolution images, and enhances the model’s generalization ability in adverse weather conditions.

Assuming an intermediate feature map *X* of size $S\times S\times C_{1}$, we use the SPD-Conv to slice the sub-feature map sequence as

(4) $$\begin{array}{cc} f_{0,0}=X\left[0:S:scale,0:S:scale\right],\; & f_{1,0}=X\left[1:S:scale,0:S:scale\right],\cdots ,\\ f_{scale-1,0}= & X\left[scale-1:S:scale,0:S:scale\right];\\ f_{0,1}=X\left[0:S:scale,1:S:scale\right], & f_{1,1}=X\left[1:S:scale,1:S:scale\right],\cdots ,\\ f_{scale-1,1}= & X\left[scale-1:S:scale,1:S:scale\right];\\ \vdots \\ f_{0,scale-1}= & X\left[0:S:scale,scale-1:S:scale\right],f_{1,scale-1},\cdots ,\\ f_{scale-1,scale-1}= & X\left[scale-1:S:scale,scale-1:S:scale\right]. \end{array}$$

where, given any (original) feature map *X*, a sub-map $f_{x,y}$ is formed by all the entries $X\left(i,j\right)$ that i+x and j+y are divisible by scale.

In the training model for multi-domain insulator defect detection and classification, we have incorporated attention mechanisms and small target detection layers to improve the model’s performance for detecting small targets. We have also optimized the model’s convolutional structure to improve accuracy without increasing computational complexity, making the structure more lightweight. We then trained the model using multi-domain insulator datasets to make it more robust in multi-domain weather conditions.

### 3.3. Weather-Domain Synthesis Module

High-voltage transmission lines operate outdoors all year round, and changing weather conditions can easily cause insulators to fail. UAV transmission line inspection can simultaneously collect multiple image data and use detection models to locate defects, saving a lot of manpower and material resources. However, most insulator defect detection models [7,11,13,29,30] only consider insulator detection under sunny weather, which is not applicable to multi-domain insulator defect detection. Therefore, we explore a novel multi-domain insulator defect detection and classification task framework based on the weather domain synthesis (WDSt) model for multi-domain insulator defect detection and classification.

Synthesizing insulator images under various weather conditions into a unified weather domain can eliminate the adverse effects of existing modal differences, simulate real complex scenarios, and improve the robustness of the insulation defect detection model to adapt to various weather conditions, thereby ensuring the accuracy and effectiveness of unmanned aerial vehicle inspection.

We designed a weather domain synthesis (WDSt) model in the testing for multi-domain insulator defect detection and classification, aiming to translate the raw insulator image into different weather-conditioned insulator images. In the work, we train a multimodal unsupervised image translation model to generate a single weather condition generator model from various weather condition insulator images, thereby reducing the detection errors caused by the multi-domain insulator modality gap. We will give more details about it as follows.

**Figure 4:**
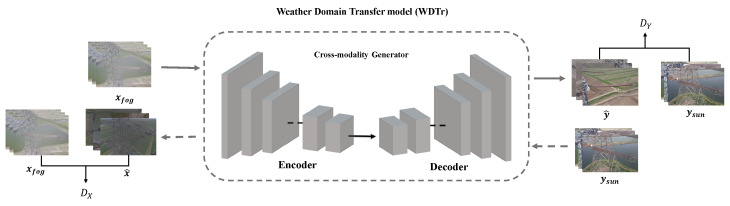
The WDSt-module converts various weather-conditioned insulator images to the uniform weather domain to decrease the domain gap.

The newly-designed Cross-modality Generator aims to translate the various weather-conditioned insulator images into uniform weather-domain insulator images. The various weather-conditioned mapping function is denoted as $G^{N}:X^{N}\to Y,N\in \{Snowy,Foggy,Rainy\}$ and $F:Y\to X^{N}$. The source domain and the target domain are represented by $X^{N}$ and *Y*, where the $X^{S}now$, $X^{F}og$, and $X^{R}ain$ mean snowy, foggy, and rainy weather insulator images, respectively, and *G* and *F* represent the two mappings. In the work, we train the single weather condition generator model for each weather. Here, we choose the foggy weather as a representative for description convenience. Inspired by related work [31,32], the data *x* from the *X* domain is passed through the generator *G* to obtain Fake $\hat{Y}$; the data *y* from the *Y* domain is passed through the generator *F* to obtain Fake $\hat{x}$. As shown in Figure 4, we are given one set of images in domain *X*, such as foggy weather insulators, and a different set in domain *Y*, such as sunny weather insulators. We may train a mapping G:X→Y such that the output $\hat{y}=G\left(x\right),x\in X$, is indistinguishable from images y∈Y by an adversary trained to classify $\hat{y}$ apart from *y*. The data *x* from the *X* domain is passed through the optimal generator *G* to obtain Fake $\hat{Y}$. Fake $\hat{Y}$ is passed through the inverse generator *F* to obtain the reconstructed result, Fake $\hat{X}$. $D_{Y}$ and $D_{X}$ are associated adversarial discriminators. $D_{Y}$ encourages *G* to translate *X* into outputs indistinguishable from domain *Y*, and vice versa for $D_{X}$ and *F*. To further regularize the mappings, we introduce two cycle-consistency losses that capture the intuition that if we translate from one domain to the other and back again, we should arrive at where we started: (1) forward cycle-consistency loss: $x\to G\left(x\right)\to F\left(G\left(x\right)\right)\approx x$; (2) backward cycle-consistency loss: $y\to F\left(y\right)\to G\left(F\left(y\right)\right)\approx y$. The objective contains two types of terms: adversarial losses [31] for matching the distribution of generated images to the data distribution in the target domain, and cycle-consistency losses [31] to prevent the learned mappings *G* and *F* from contradicting each other.

The original adversarial loss formula is as follows:

(5) $$\begin{array}{cc} L_{GAN}\left(G,D_{Y},X,Y\right) & =E_{y∼p_{data\left(y\right)}}\left[logD_{Y}\left(y\right)\right]\\ \; & +E_{x∼p_{data(x)}}\left[log{(1-D}_{Y}\left(G(x)\right)\right], \end{array}$$

where *G* tries to generate images $G\left(x\right)$ that look similar to images from domain *Y*, while $D_{Y}$ aims to distinguish between translated samples $G\left(x\right)$ and real samples *y*. *G* aims to minimize this objective against adversary *D* that tries to maximize it. We introduce a similar adversarial loss for the mapping function F:Y→X and its discriminator $D_{X}$ as well.

The image *x* from domain *X* and the image translation cycle should be able to bring *x* back to the original image, such as the forward cycle-consistency loss: $x\to G\left(x\right)\to F\left(G\left(x\right)\right)\approx x$; similarly, the image *y* from domain *Y* as well. For the mechanism to train stably, a cycle-consistency loss formula needs to be calculated as follows:

(6) $$\begin{array}{cc} L_{cyc} & =E_{x∼p_{data\left(x\right)}}\;\left[{∥F\left(G\left(x\right)\right)-x∥}_{1}\right]\\ \; & +E_{y∼p_{data\left(y\right)}}\;\left[{∥G\left(F\left(y\right)\right)-y∥}_{1}\right]. \end{array}$$

The final loss formula is as follows:

(7) $$\begin{array}{cc} L_{final}\left(G,D_{Y},F,D_{X},\right) & =E\left[logD_{Y}\left(y\right)\right]+E\left[log{(1-D}_{Y}\left(G(x)\right)\right]\\ \; & +E\left[logD_{X}\left(x\right)\right]+E\left[log{(1-D}_{X}\left(G(Y)\right)\right]\\ \; & +\lambda L_{cyc}. \end{array}$$

Here, λ controls the relative importance of the *G* and *F*, which means that the generator *G* should achieve the transfer from *X* to *Y* as much as possible, and the generator *F* should achieve the transfer from *Y* to *X* as much as possible. At the same time, it is hoped that the two generators can achieve reciprocity, that is, they can iteratively return to themselves. We only select the *G* as the cross-modality insulator generator model, which can translate the raw images into different weather-conditioned insulator images.

### 3.4. The Evaluation Indicator System in the Insulator Defect Detection Model

Common metrics for object detection accuracy include Precision (Precision), Recall (Recall), Average Precision (AP), Mean Average Precision (mAP), Intersection over Union (IoU), and Precision–Recall Curve. In the experimental results presented in Section 4 of this article, three evaluation metrics were used: Precision, Recall, and Mean Average Precision (mAP). The calculation formula is as follows:

(8) $$\begin{array}{c} P=\frac{N_{TP}}{N_{TP}+N_{FP}},\\ R=\frac{N_{TP}}{N_{TP}+N_{FN}}, \end{array}$$

where $N_{TP}$ is the number of correctly predicted positive samples; $N_{FP}$ is the number of incorrectly predicted positive samples; $N_{TN}$ is the number of correctly predicted negative samples; and $N_{FN}$ is the number of incorrectly predicted negative samples. In target detection algorithms, there are many evaluation metrics.

The mean of all AP for each class in the dataset is taken to obtain mAP:

(9) $$\begin{array}{cc} AP & =\frac{1}{m}\sum_{i}^{m}P_{i}\\ \; & =\frac{1}{m\;}*P_{1}+\frac{1}{m\;}*P_{2}+\ldots +\frac{1}{m\;}*P_{m}\\ \; & =\int P\left(R\right)dR\;, \end{array}$$

where *R* is recall, and *P* is precision. AP is the average precision for a certain class of n samples; assuming it has *m* positive examples, each positive example corresponds to a Recall value $\left(\frac{1}{m},\frac{2}{m},\cdots ,1\right)$, and the maximum Precision is calculated for each recall. Then, the mean of these Precision values is taken. The mean of all AP for each class in the dataset is taken to obtain mAP:

(10) $$mAP=\frac{1}{C}\sum_{j}^{C}{AP}_{j},$$

where *P* is precision, AP is the average precision of a class of samples, and mAP is the average precision of the dataset. The mAP@50 represents the mAP values with an IoU of 0.5.

## 4. Experiments

In this section, we evaluated the proposed multi-domain insulator defect detection on our proposed multi-domain insulator databases (MD-insulator). We compared other popular methods and the experimental results prove that our method achieved a satisfactory performance in the multi-domain insulator defect detection and classification task. Then, we investigate the effect of different parameters on the recognition performance. Finally, we conduct the ablation study to evaluate the effectiveness of the proposed WDSt and CIA-Yolo modules.

### 4.1. Databases

The currently available public datasets, such as the CPLID dataset [20] for insulator detection, are based on images of single-domain insulators and one type of self-exploding insulator defect, which cannot fully reflect the sample insulator characteristics of power lines. The insulator defect detection model trained using this dataset is only applicable to specific weather conditions and defect types and is not suitable for multi-domain insulator defect detection and classification in real-world complex scenarios with multiple defects and various weather conditions. Zhang et al. [17] proposed a dataset for insulator detection in foggy weather conditions. This dataset contains 853 original images and 10,122 augmented images total, which are augmented with random masking, random left and right flips, random up and down flips, etc.

**Figure 5:**
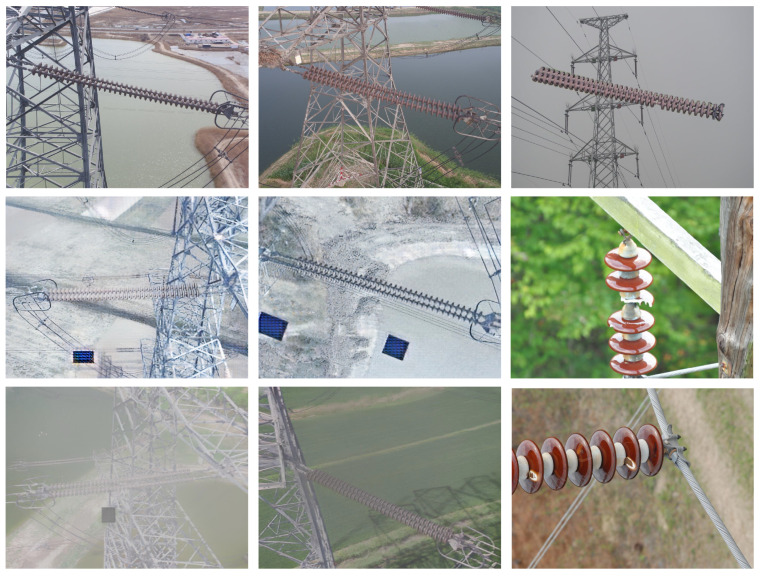
The multi-domain insulator dataset (MD-Insulator) contains 12,605 insulator images with three common insulator defects: self-explosion, flashover damage, and broken insulator.

Therefore, in the following experiment, we constructed a new *multi-domain insulator dataset (MD-Insulator)* for defect detection and classification. The MD-insulator dataset is almost fully collected from the public SFID dataset [17], and the rest is collected by individuals. There are a total of 16,430 insulator images, including 5318 images of insulator defects, with defect categories including self-explosion, flashover damage, and broken insulator. The example insulator images are shown in Figure 5. The image resolutions are 1152×864, 2144×1424, and 2136×3216, and the training set, validation set, and test set of the network model were trained according to a ratio of 7:2:1.

### 4.2. Implementation Details

We utilize the YOLOv8 model as the backbone network in the multi-domain insulator defect detection and classification module. This method is implemented based on the deep learning framework PyTorch and accelerated using an Nvidia RTX 3060 GPU. The model is trained in a limited 200 epochs with a batch size of 16 and a learning rate of 0.001.

In the WDSt module, we trained from scratch using the Adam solver [31] with a batch size of 1 and a learning rate of 0.0002. We keep the same learning rate for the first 100 epochs and linearly decay the rate to zero over the next 100 epochs. In the cyclic consistency loss (Section 3.3), we set λ=10 in Formula (7). Specifically, in the test model, we first use the WDSt module to convert insulator images under diverse weather conditions (e.g., snowy, rainy, and foggy weather) into a unified weather domain (sunny weather) for insulator defect detection and classification using the Cross-modality Information Attention YOLO model, to decrease the existing domain gap and improve the detection capability for multi-domain insulator defects.

### 4.3. Comparison Experiment

To verify the effectiveness of the proposed method, we conducted comparative experiments using different detection models under the same conditions, as shown in Table 1. All models were trained using the MD-Insulator dataset and tested in foggy and snowy weather conditions. It can be seen that our model is more likely to identify insulator defects under multi-domain weather conditions. The flashover damage defect that is not easy to identify, the Precision metric, increased from 66.8% to 80%; the mAP@50 metric increased from 28.1% to 79%; the broken insulator defect mAP@50 metric increased from 41.7% to 74.9%; and the Precision metric increased from 59% to 85.4%. The results indicate that the rate has been improved using the proposed method in this paper, which can effectively improve the recognition rate of insulators under multi-domain weather conditions, especially for small targets and difficult-to-recognize flashover damage and broken insulator defects, and is more valuable in the real world.

For the convenience of comparison, we used the official DETR [36], Yolov5 [34], and Yolov8 [35] models. The method is implemented based on the PyTorch platform and Nvidia RTX 3060 GPU. The results of the mAP@50 metric and the mAP@50:95 metric for the YOLOv5 model [34] are 84.6% and 56.3%, respectively; the results for the YOLOv8 model [35] are 80.1% and 55.4%, respectively; the results for the DETR model [36] are 82.3% and 50.6%, respectively; and the results for the proposed model are 88.3% and 62.6%, respectively. From the results, it can be seen that the DETR model does not perform well on the multi-domain insulator defect detection task. It may be that DETR [36] does not pay special attention to the detection of small objects during training, leading to poor performance on small targets such as broken insulators and flashover damage. The proposed method in this paper achieves 88.3% on the mAP@50 metric, which is 5.5% higher than the DETR model, and also improves by 12% on the mAP@50:95 metric. Therefore, it can be concluded that our model has good detection ability in the multi-domain insulator defect detection task.

**Figure 6:**
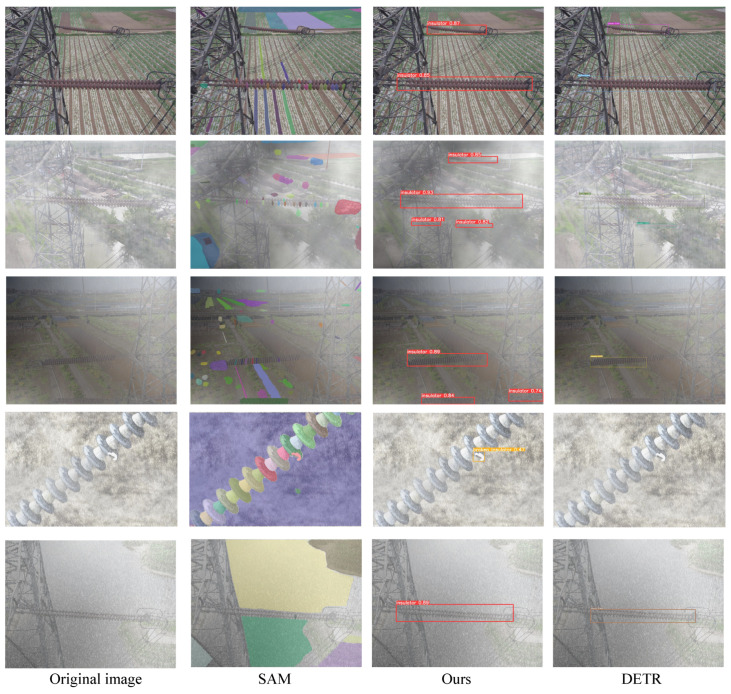
The detection results of the multi-domain insulator defect detection and classification model.

In the multi-domain insulator defect detection task, the detection is more suitable than segmentation. (1) The image segmentation task depends on the continuity of pixel points, and the algorithm relies on the accuracy and quality of the input image. For low-quality or blurred images, the segmentation effect may be affected. In the multi-domain insulator detection task, foggy, rainy, and snowy weather conditions can interfere with pixels, resulting in differences in segmentation boundaries and leading to misclassifications. (2) Compared with segmentation, the detection model only needs to detect anchor points which is a more accessible approach, especially for small targets, occlusion, and adverse weather conditions. We find that the instance segmentation does not fit the problem. Here, we choose SAM [37] as the representative segmentation method, the DETR [36] model, and the proposed method for comparison in the multi-domain insulator defect detection task, as shown in Figure 6.

From the comparison results, we can conclude that the SAM segmentation algorithm performs poorly in multi-domain insulator detection tasks, especially in multi-target and multi-scale insulator images where it often fails to segment all insulator targets. For clear photos of insulator defects (as shown in column 4), although SAM can segment the outline of the insulator, it requires manual verification of the defect category, which can be a labor-intensive task. The DETR detection method fails to detect small target insulators in the distance and small target defects in multi-domain insulator detection tasks. It is possible that DETR does not pay special attention to the detection of small objects during training, resulting in poor performance for small objects and inaccurate detection results. Thus, both SAM and DETR algorithms do not have advantages in multi-domain insulator detection tasks.

### 4.4. Ablation Study

The proposed weather domain synthesis network for extracting cross-modality discriminative information on multi-domain insulator defect detection and classification framework mainly contains two modules of our design: the weather domain synthesis (WDSt) module and the Cross-modality Information Attention YOLO (CIA-YOLO) module. To reveal how each module contributes to performance improvement, we conduct a comprehensive ablation study to analyze them on the MD-insulator dataset as shown in Table 2 and Table 3.

We selected five different attention mechanisms for ablation experiments and obtained the best-performing CIA-YOLO model. Among them, the SE attention [24] (Squeeze-and-Excitation Networks) focuses on channel relationships, adaptively recalibrating channel feature responses by explicitly modeling the interdependencies between channels. The CA [25] (Coordinate Attention) optimizes the position information ignored by SEnet [24] in visual tasks, embedding position information into the attention mechanism and capturing long-range dependencies in one spatial direction while maintaining accurate position information in another spatial direction. The ECA [27] (efficient channel attention) module only adds a small number of parameters but can achieve significant performance gains. The CBAM [26] (Convolutional Block Attention Module) is a combination of spatial and channel attention mechanism modules. Compared to SEnet [24], which only focuses on channel attention, CBAM [26] can achieve better results. The SA [23] (Shuffle Attention) effectively combines two types of attention mechanisms, spatial attention and channel attention, using Shuffle units to achieve better performance while avoiding computational overhead. To improve the detection accuracy of small targets, we also added extra small target detection layers and then connected feature maps of different scales in a pyramid form to fuse high-level and low-level features. In the FPN (Feature Pyramid Network) network, we introduced the weighted bidirectional feature pyramid BiFPN [21] to enhance the low-level information of the feature map, enabling information fusion of feature maps of different scales and thereby strengthening feature information.

Table 2 and Table 3 summarize the performance of the proposed variants of the method. We used the pure YOLOv8n algorithm as the baseline method and trained it using the MD insulator dataset for fair comparison. Due to the limited weather mode gap in the insulator dataset, the baseline performance of the insulator defect detection model task is poor. Table 2 shows the experimental results using our CIA-YOLO strategy. The Precision metric of the detection model increased from 79.6% to 90.5%, and the mAP@50 metric increased from 74.1% to 88.2%, achieving the best recognition performance for insulator defects. This is thanks to the CIA-YOLO algorithm, which utilizes attention mechanisms to add detection layers in the network head specifically for small targets, improving the model’s ability to detect small defects in insulators. We used an adverse weather conditions’ test set to validate the performance of the WDSt model, and the results are shown in Table 3. Although it performed poorly in identifying flashover defects (surface defects) in extreme weather, its ability to identify insulators performed well. The Precision metric of the detection model increased from 86.4% to 89.1%, and the mAP@50 improved by 17.3%, from 68.6% to 85.9%. This is because the WDSt model we designed reduces the domain gap between different weather conditions for multi-domain insulators.

### 4.5. Cross-Dataset Evaluation

The analysis of the ablation experiment in the previous section shows that the cross-modality discriminative information with the weather domain synthesis model not only makes the detection model more robust, but also improves the overall performance. In this section, we test the performance of the proposed method on the SFID [17] and CPLID [20] test sets. The SFID is a dataset that includes insulators under foggy weather conditions, where the test set contains 4318 insulators and 760 self-detonation defects. The CPLID dataset consists of insulators captured by UAV under sunny weather conditions, including 600 normal insulators. These models are trained in a limited 100 periods.

The performance of our proposed method on different datasets is shown in Table 4. The results indicate that our method performs better in multi-domain insulator weather conditions, especially for detecting small tasks. On the test set SFID, the Precision, Recall, and mAP@50 scores for self-explosion defects are 99.6%, 99.1%, and 99.5%, respectively. The experimental results demonstrate that the proposed multi-domain insulator defect detection algorithm can achieve satisfactory recognition performance on other test sets as well.

### 4.6. Algorithm Analysis

**Figure 7:**
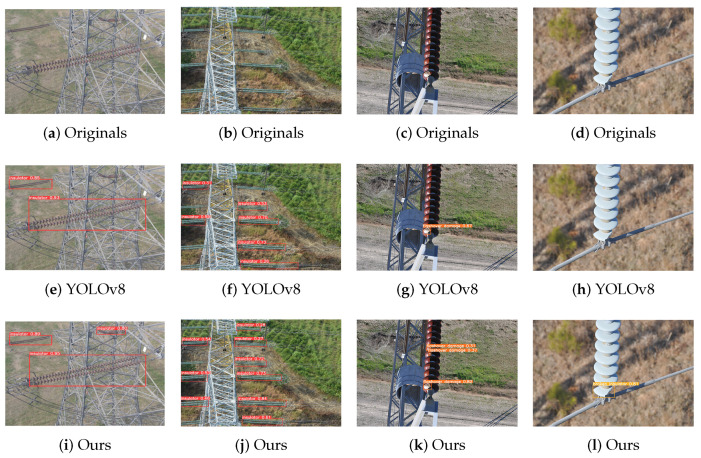
The detection results of the multi-domain insulator defect detection and classification model.

Insulators operate outdoors and are deeply affected by weather changes, leading to faults. In complex environments, the targets of insulator defects are small and easily concealed, especially flashover damage and broken insulator defects that are difficult to detect. Therefore, if a model can accurately identify defects in complex scenarios, it is crucial to ensure the safe operation of electrical power. In addition, we tested the detection results of four groups of insulator models in different scenarios, shown in Figure 7. The first one tested the model’s ability to detect insulators under foggy weather conditions; the second one tested the model’s ability to detect multiple targets in remote views; and the last two are the ability to detect flashover damage and broken insulator defects that are difficult to identify. The four sets of detection results in Figure 7 show that the detection results of this method are superior to those using the original YOLOv8. In foggy conditions, using the original YOLOv8 means that it cannot detect insulators that are obscured by towers. In the distant view with complex scenarios, the detection model proposed in this paper can detect more insulator strings, as the detection results of flashover damage and broken insulator defects show that the proposed method can not only detect more comprehensive flashover damage, but also has a more sensitive detection of broken insulator defects.

**Figure 8:**
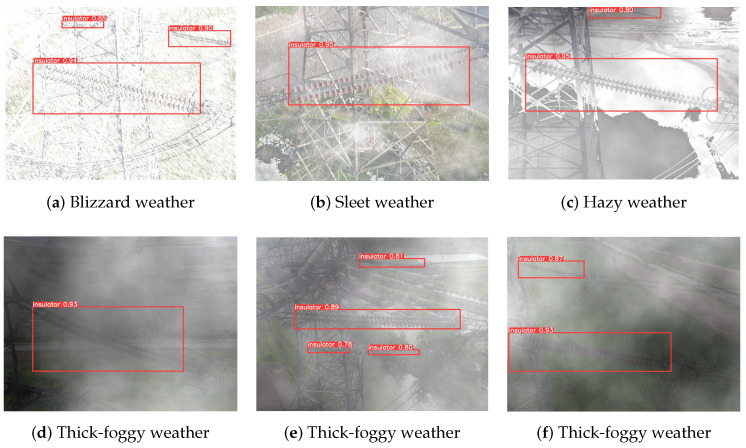
The results of the insulator defect detection and classification model in extreme weather.

To verify the model’s ability to detect insulators under extreme weather conditions, we simulated a set of insulator images under different levels of weather complexity, and the detection results are shown in Figure 8.

The above test results show that using the multi-domain insulator defect detection and classification model proposed in this paper has the best detection performance, especially in remote and multi-task scenarios of insulator detection in unmanned aerial vehicles (UAVs) power transmission line inspection, overcoming the impact of weather changes on the model detection ability. Therefore, the proposed detection model can output relatively reliable results regardless of the distance, angle, and weather conditions of the insulator in the detected image of the UAV and has strong robustness and generalization capabilities.

## 5. Conclusions

Insulators are important measures to ensure the safe and stable operation of power transmission lines, and they are prone to damage under variable weather conditions. Therefore, the insulator defect detection model for UAV inspection needs to have good generalization ability to adapt to multi-domain insulator defect detection and classification. In this paper, we propose a weather domain synthesis network for extracting cross-modality discriminative information on multi-domain insulator defect detection and classification tasks. This paper explores a novel weather domain synthesis module (WDSt) for multi-domain insulator defect detection tasks, which gives a new perspective to decreasing the multi-domain insulator modality gap in diverse weather conditions. The proposed Cross-modality Information Attention YOLO (CIA-YOLO) module aims to utilize attention mechanisms and add a detection layer in the network head to improve the model’s ability to detect defects in multi-domain insulators. The experimental results on the proposed multi-domain insulator dataset (MD-Insulator) illustrate the superior performance of the proposed method compared with other methods. In the future, we will evaluate the proposed method’s performance on more complex multi-domain insulator datasets and explore better robustness to mimic real-world scenarios.

## Figures and Tables

**Table 1:** A comparison of the multi-domain insulator defect detection and classification algorithms.

Model	Class	Precision (%)	mAP@50 (%)
Faster RCNN [33]	Insulator detection	-	88.6
	Self-explosion	-	85.4
	Flashover damage	-	43.1
	Broken insulator	-	53.5
YOLOv5 [34]	Insulator detection	95.0	95.6
	Self-explosion	92.1	96.0
	Flashover damage	72.0	65.4
	Broken insulator	71.5	61.2
YOLOv8 [35]	Insulator detection	94.1	98.7
	Self-explosion	98.3	99.5
	Flashover damage	66.8	56.7
	Broken insulator	59.1	41.7
Ours	Insulator detection	**97.0**	**99.2**
	Self-explosion	**99.6**	99.5
	Flashover damage	**80.1**	**79.0**
	Broken insulator	**85.4**	**74.9**

**Table 2:** The ablation study adds different attention mechanisms and extra detection layers in the network, where the baseline utilizes a pure YOLOv8n model.

Baseline	SA	CBAM	ECA	BiFPN	Ours	Precision (%)	Recall (%)	mAP@50 (%)
✓	-	-	-	-	-	79.6	71.2	74.1
✓	✓	-	-	-	-	87.1	70.6	76.3
✓	-	✓	-	-	-	86.1	72.0	77.1
✓	-	-	✓	-	-	81.1	73.4	76.8
✓	-	-	-	✓	-	85.7	74.2	79.2
✓	-	-	-	-	✓	**90.5**	**82.6**	**88.2**

✓ means the module utilized in the experiment.

**Table 3:** The ablation study on the WDSt model and the CIA-YOLO model, where the baseline utilizes a pure YOLOv8n model.

Baseline	WDSt	CIA-YOLO	Precision (%)	Recall (%)	mAP@50 (%)
✓	-	-	86.4	63.5	68.6
✓	✓	-	**89.1**	**83.6**	**85.9**

✓ means the module utilized in the experiment.

**Table 4:** Cross-database testing accuracies (%) of the proposed approach using CPLID and SFID.

Train Set	Test Set	Classes	Number	Precision (%)	Recall (%)	mAP@50 (%)
MD-insulator	CPLID	Insulator	1073	97.1	97.3	99.1
	SFID	Insulator	4318	96.2	96.6	99.0
	SFID	Defect	760	99.6	99.1	99.5

## Data Availability

The Chinese Power Line Insulator Dataset (CPLID) can be downloaded at https://github.com/InsulatorData/InsulatorDataSet, accessed on 30 December 2023; The synthetic foggy insulator dataset (SFID) can be downloaded at https://github.com/zhangzhengde0225/FINet, accessed on 31 December 2023.
